# A novel experimental setup for evaluating the stiffness of ankle foot orthoses

**DOI:** 10.1186/s13104-018-3752-4

**Published:** 2018-09-05

**Authors:** A. Ielapi, E. Vasiliauskaite, M. Hendrickx, M. Forward, N. Lammens, W. Van Paepegem, J. P. Deckers, M. Vermandel, M. De Beule

**Affiliations:** 1 Institute Biomedical Technology (IBiTech)-bioMMeda, Ghent University, Corneel Heymanslaan 10, Block B, 9000 Ghent, Belgium; 2 Gait & Movement Analysis Laboratory, Cerebral Palsy Reference Centrum, University Hospital Ghent, 9000 Ghent, Belgium; 3 Department of Materials Science & Engineering, Ghent University, Technologiepark-Zwijnaarde 903, 9052 Zwijnaarde, Belgium; 4 SIM vzw, Technologiepark 935, 9052 Zwijnaarde, Belgium; 5 V!GO NV, Biezeweg 13, 9230 Wetteren, Belgium

**Keywords:** Experimental setup, Ankle foot orthosis (AFO), Stiffness, Gait, Orthotics

## Abstract

**Objective:**

The purpose of this study was the construction of a new semi-automated experimental setup for the evaluation of the stiffness of ankle foot orthoses (AFOs) around an axis aligned to the anatomical ankle joint during the second rocker of the gait. The setup, developed in close collaboration with the orthopedic device company V!GO NV (Wetteren, Belgium), allows measurement of plantarflexion and dorsiflexion in the sagittal plane for a maximal range of motion of 50° (− 25° plantarflexion up to 25° dorsiflexion) in a non-destructive way.

**Results:**

The mechanical properties of four 3D printed AFOs are investigated, based on the ranges of motion derived from the gait assessment of the patients when they walked with their AFO. The reliability of the stiffness measures was studied by the evaluation of the test–retest repeatability and the intra-tester and inter-tester variability. These studies revealed that the ankle stiffness can be measured with high reliability (ICC = 0.94–1.00). The obtained outcomes indicate that the experimental setup could be applied to measure the ankle stiffness of any topology of AFOs and, in the future, help finding the correlation with the information coming from the gait assessment of the patients.

**Electronic supplementary material:**

The online version of this article (10.1186/s13104-018-3752-4) contains supplementary material, which is available to authorized users.

## Introduction

Ankle foot orthoses (AFOs) are external medical devices, applied around the ankle joint, to provide support and stability for weakened muscles, proper control of the limbs and protection [1–3]. Their impact depends on the properties of the material used and the design: for obtaining the optimal functional gains it is essential to customize the AFOs to the patient needs [4]. Currently, the most used AFOs are custom-molded thermoplastic AFOs [5], which provide the patient with an intimate fit. A critical role is played by the craftsmen, who directly manufacture the devices, in a process which is manual and time consuming [6, 7]. However, this manufacturing process does not allow modifications of the design parameters before the realization of the devices. Researchers are currently focusing on the use of new additive manufacturing (AM) techniques, which should permit the tuning and optimization of the AFOs mechanical properties [8, 9]. In addition, the.stl files used for 3D printing, which describe the geometry of the AFOs, could be used for the creation of finite element models in order to predict their mechanical properties [10]. From a clinical point of view, AFO stiffness represents a key factor: it determines how the gait will be influenced by adding a certain contribution to or against the action of the patient’s muscles [11]. Several studies have used experimental setups for measuring AFO stiffness along different ranges of motion: some of them focused on the quantification in the sagittal plane by manual [12–16] or automated control [17]; Cappa et al. [11] developed a manual control setup to assess the mechanical properties in both sagittal and frontal planes; and some year after developed an automated loading apparatus for experiments in the three different directions [18]; Klasson et al. [19] investigated similar quantities but with a manually controlled system; Bregman et al. [20], instead, created a manual controlled apparatus which allows not only the evaluation of the AFO stiffness in the sagittal plane around the ankle joint, but also around the metatarsal-phalangeal (MTP) joint. Yamamoto et al. [21] measured AFO properties in vivo by placing the patient’s AFO and foot in a muscle training machine, while Polliack [22] created a test rig able to simulate the AFO behavior during the three phases of the gait cycle. Unfortunately, the reliability of these devices is not always defined and most of them are controlled manually, which makes it difficult to test the AFOs in a controlled manner.

Therefore, the aim of this study is to describe the design and the application of a new semi-automated experimental setup which evaluates the stiffness of AFOs in a reliable non-destructive way, around an axis aligned with the anatomical ankle joint during the second rocker of the gait.

## Main text

### Methods

**Fig. 1:**
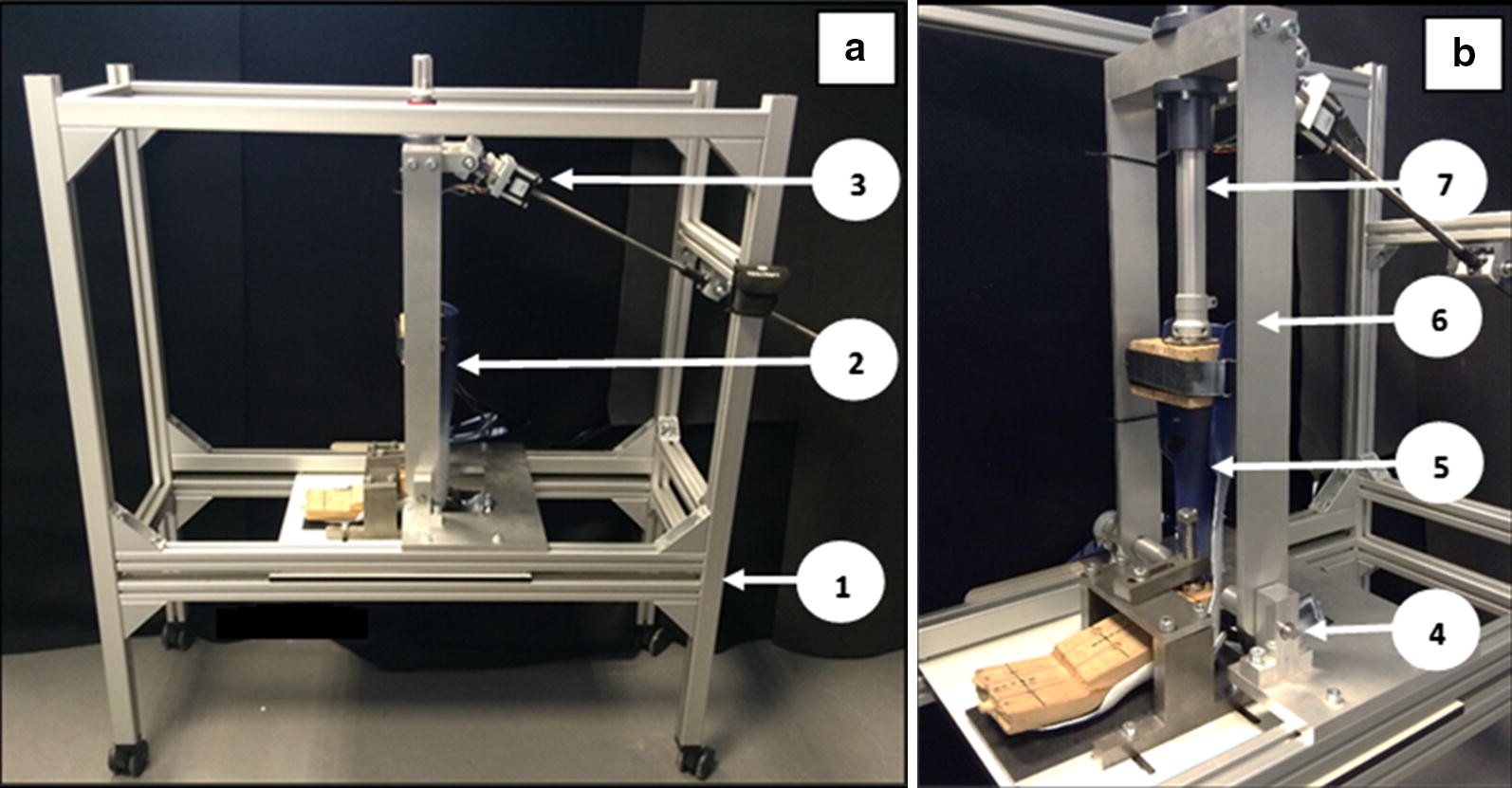
Overview of the experimental setup for testing the 3D printed AFOs: (1) external frame; (2) AFO; (3) linear Motor; (4) ankle rotation axis; (5) closer view of the clamped AFO; (6) U-shaped frame; (7) shank axis

The key design specification for the experimental setup was to be able to measure the stiffness of a wide variety of AFOs over patient-specific ranges of motion in the sagittal plane during the second rocker of the gait (Fig. 1a, b).

The design allows measurement of stiffness around an axis aligned with the anatomical ankle joint: this is achieved by aligning the anatomical ankle joint, present on a model of the patients leg, with the rotation axis of the setup. The model of the leg is milled from medium-density fibreboard (MDF) and contains anatomical landmarks of the patient related to his/her gait assessment. Small surface markers are placed on the patient’s medial and lateral malleoli just prior to digital scanning of the leg. The STL file derived from the scan and used to mill the MDF model contains the anatomical references required for the alignment in the test rig (Additional file 1).

After milling, the MDF block is cut in three parts: a calf, an ankle and a foot part. The calf part is used for the connection with the shaft of the setup, which represents the shank axis, and is strapped to the AFO. The ankle part is only used for ensuring the alignment of the AFO during the clamping in the test rig. The test rig is designed with two pointers which facilitate the alignment at the ankle axis (Additional file 2, item 3). The foot part is used for clamping the sole section of the AFO: a compression screw clamps the AFO sole section between the MDF foot section and the test rig base plate in a non-destructive manner (Additional file 2, item 2).

With the AFO mounted, plantarflexion and dorsiflexion can be applied to the orthosis: dorsiflexion is the movement of the AFO calf towards the foot section, while plantarflexion is the reverse movement. The rig design allows up to 25 degrees in both dorsiflexion and plantarflexion, respectively indicated with positive and negative angles, from an initial neutral angle of 0 degrees. The U-shaped frame connects the shaft of the shank axis with the ankle rotation axis (Fig. 1b). A linear motor (Haydon™ Size 23) with a spindle length of 750 mm drives the rotation of the U-shaped frame around the ankle rotation axis. At the same time the shaft can slide up and down through the presence of two bearings, which prevent excessive loading on the AFO.

The stiffness around the ankle joint is defined as the moment around the ankle joint exerted by the AFO per degrees of ankle joint rotation [20]. The ankle rotation is recorded by an incremental optical encoder (Kubler™ 5020), which is positioned around the ankle rotation axis of the test rig (Additional file 2, item 4). The moment is recorded by a load cell (Sensy™ 2712) located behind the linear motor, which allows the acquisition of the force acting on the AFO (Additional file 3). Before the experiment, the neutral angle, which represents the configuration of the AFO when no external moment is applied [20] is also measured, by using a digital goniometer (Toolcraft 816141). For every range of motion, a calibration curve, recorded when the AFO is not inserted in the setup, is required to eliminate the gravitational effects given by the weight of the MDF blocks and the hardware of the setup.

**Fig. 2:**
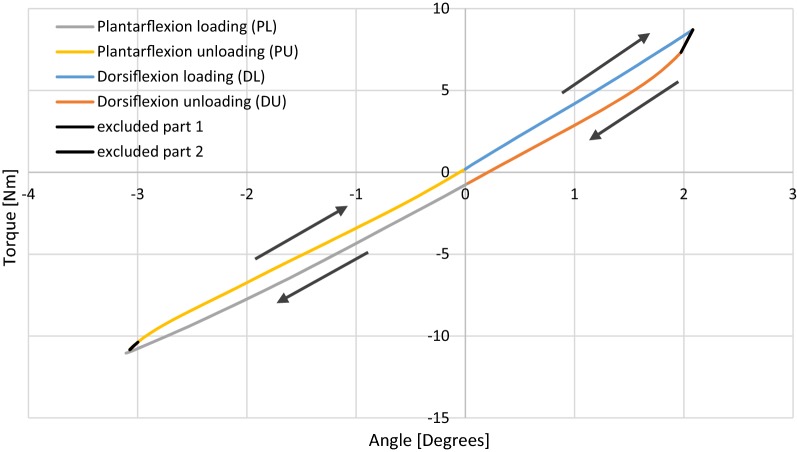
Example of the stiffness calculation in the four quadrants: plantarflexion loading (PL), plantarflexion unloading (PU), dorsiflexion loading (DL), dorsiflexion unloading (DU) and the excluded parts 1 and 2

Control, data collection and visualization are carried out using a custom written LabView code. Post-processing is done with a dedicated Python script, which permits the calculation of the moments starting from the forces acquired with the load cell. The script allows the calculation of the stiffness, by linear fitting, in four different quadrants: plantarflexion loading (PL), plantarflexion unloading (PU), dorsiflexion loading (DL) and dorsiflexion unloading (DU) (Fig. 2). Since the operational speed of the linear actuator is constant, going from dorsiflexion to plantarflexion and vice versa cannot be instantaneous, but requires a certain time depending on the de/acceleration. The data gathered during de/acceleration was excluded from further use (Fig. 2). During the measurement, five cycles are recorded plus one calibration curve at a speed of 1 degree/s. A 4th order Butterworth filter with a cut-off frequency of 0.2 Hz was used to filter the data, while the sampling frequency was 10 Hz.

In this study, four 3D printed AFOs were measured, for patients with a EU foot size of 32, 35, 37 and 45, respectively indicated as AFO A, B, C and D. All the AFOs are made of three parts [23]: a foot and a calf part made in Polyamide 12 (PA 12) connected by two carbon rods (6 mm diameter for AFOs A, B and C; 8 mm for AFO D). The range of motion for the calculation of the stiffness was derived from gait assessment data obtained when the patients walked with their AFO: − 2°/6° for AFO A, − 6°/3° for AFO B, − 6°/10° for AFO C and − 7°/9° for AFO D.

By performing a series of tests on the four AFOs, the influence of different sources of error were investigated: AFO A, B, C and D were used to investigate the test–retest repeatability of the test rig excluding the AFO mounting process (using three repeated measures by the same operator with the AFO remaining fastened inside the setup between test re-test trials), the intra-tester variability (variability when the device is removed and reinserted in the setup by the same operator on different days) and the inter-tester variability (measures on the same device by two different operators with removal of the AFO from the test rig between repeat tests).

In order to test the reliability of the stiffness measures the Intraclass Correlation Coefficient (ICC) is calculated by using a two-way mixed-effects model in SPSS 24.0 [24]. The standard error of measurement (SEM), as an indication of the expected measurement error in a single individual score, can be also calculated, by multiplying the standard deviation (SD) of the measures with the square root of (1-ICC). Then the Smallest Detectable Difference (SDD), calculated as the SEM multiplied for 1.96 and the square root of 2, defines a threshold value of change in scores for the tester to be 95% confident that true change beyond that of measurement error had occurred [19].

Prior to measuring the AFOs, a test object ‘CalibrAFO’ was designed, for testing an inox steel sheet inside the test rig. In order to validate these results, a calibration of the elastic properties of the inox sheet was performed with a “Instron Electropuls E10000” machine (Additional file 4).

### Results

The results of the experimental tests on the inox steel sheet coming from the experimental setup and the Instron testing machine showed comparable outcomes with differences due to the different clamping conditions (Additional file 5).

In terms of the statistical analysis, the test–retest repeatability reveals that the maximal percentage difference is never higher than 2% (Additional file 6). Concerning the intra-tester analysis, the percentage difference is not higher than 5.26% (Additional file 7), while for the inter-tester variability the error is not higher than 5.65% (Additional file 8). The calculation of the ICC (Table 1) shows high reliability with values ranging from 0.94 to 1. In addition, the SEM expresses relatively low values of measurement error, while, the SDD provides the system discrimination.

**Table 1:** Calculation of ICC, SEM and SDD

	PL			PU			DL			DU		
	ICC (%)	SEM (Nm/°)	SDD (Nm/°)	ICC (%)	SEM (Nm/°)	SDD (Nm/°)	ICC (%)	SEM (Nm/°)	SDD (Nm/°)	ICC (%)	SEM (Nm/°)	SDD (Nm/°)
Test–retest	1	0	0	1	0.25	0.69	1	0.06	0.16	0.99	0.09	0.24
Intra-tester	0.99	0.29	0.81	0.97	0.40	1.10	0.97	0.19	0.53	0.94	0.25	0.70
Inter-tester	0.99	0.19	0.54	0.98	0.29	0.80	0.94	0.25	0.69	0.98	0.12	0.32

### Discussion

This study presents a new semi-automated experimental test rig for the evaluation of the stiffness of AFOs around an anatomically aligned ankle axis over a maximum range of ± 25 degrees, during the second rocker of the gait. This study focused on 3D printed orthoses but potentially the test rig can be applied to any topology of AFOs, since it gives the possibility to accommodate a wide range of AFOs, which are secured by using a patient-specific leg model. This leg model contains the location of the anatomical points defining the ankle flexion/extension axis used in the gait analysis and the rig applies moments around it to derive the AFO stiffness. The four AFOs, used in this study, were tested over a patient-specific range of motion according to the data coming from the gait analysis to ensure the best approximation of the AFO stiffness felt by the patient during the second rocker of the gait.

#### Reliability of the setup

Different factors were studied: the test–retest repeatability, the intra-tester and inter-tester variability. For the test–retest repeatability, the maximal error is never higher than 2%. Schrank et al. [14], evaluated the test–retest variability for 3D printed AFOs obtaining a maximal difference of 4.7%, but only considering two repetitions. Other studies [20, 25] assessed the test–retest variability by calculating the ICC on thermoplastic and/or carbon fiber AFOs, obtaining good results as in our study (Table 1). Bregman et al. [20] also reported high reliability in terms of the intra-tester and inter-tester variability, similar to the values we obtained. In addition, the calculation of the SEM revealed low values of measurement error associated to each variability index (Table 1).

#### Hysteresis

All the stiffness plots showed the presence of hysteresis (Fig. 2), which is dependent upon the strain rate employed to deform the devices [26] and by the friction present between the AFO and the test rig and between the components of the test rig. In contrast with other authors [11–22], four different ankle stiffness values are considered for each zone of the angle vs. torque curve, as it can be observed that most patient-specific AFOs have a different behaviour in plantarflexion compared to dorsiflexion due to their shape. Hysteresis may lead to an overestimation of the measured stiffness in the unloading phases, especially if high ranges of motion are used.

### Conclusions

Overall, the obtained results indicate that the experimental setup is able to quantify the stiffness values of the AFOs over their specific ranges of motion in a non-destructive manner and that could be applied to find the correlation with the information coming from the patients’ gait assessment.

## Limitations

Because of the current instrumentation, the influence of speed on the AFOs was not investigated; modifications will be applied to analyze its effect. Because of its viscoelastic properties, the direct use of a human limb might have an impact on the stiffness measures and provide a better representation of the anatomical movement at the ankle. Further studies will be performed in the future.

## Additional files

**Additional file 1.** Description and graphical representation of the anatomical points on the MDF blocks.

**Additional file 2.** Detail of the experimental setup.

**Additional file 3.** Detail of the load cell–linear motor complex.

**Additional file 4.** Figure of the two experimental settings used to test the ‘CalibrAFO’ device.

**Additional file 5.** Results obtained from the tests on the ‘CalibrAFO’ device.

**Additional file 6.** Results in terms of the AFO rig test–retest repeatability.

**Additional file 7.** Results in terms of the AFO rig intra-tester variability.

**Additional file 8.** Results in terms of the AFO rig inter-tester variability.

## Abbreviations

AFO: ankle foot orthosis
AM: additive manufacturing
MTP: metatarsal–phalangeal
MDF: medium-density fibreboard
PL: plantarflexion loading
PU: plantarflexion unloading
DL: dorsiflexion loading
DU: dorsiflexion unloading
PA 12: polyamide 12
ICC: Intraclass Correlation Coefficient
SEM: standard error of measurement
SD: standard deviation (SD)
SDD: small detectable difference
