# Supplementary material for: A novel experimental setup for evaluating the stiffness of ankle foot orthoses
Source: BMC Res Notes. 2018 Sep 5;11:649. doi: 10.1186/s13104-018-3752-4 (PMC6125880; doi:10.1186/s13104-018-3752-4)
Supplement: Supplementary file 1 — Additional file 1. Description and graphical representation of the anatomical points on the MDF blocks. [file 13104_2018_3752_MOESM1_ESM.docx]

**Description of the anatomical points on the MDF blocks**

The model of the leg is milled from medium-density fibreboard (MDF) and contains anatomical landmarks of the patient related to his/her gait assessment. The objective being to make a direct connection between the kinematics as defined and measured in the gait laboratory when walking both barefoot and with AFO, in mechanical testing of the AFO and in computer simulations. In the kinematic modelling (which is based on an adapted version of Plug-in-Gait from VICON Motion Systems), the knee joint centre is defined as the point between the centre of the medial collateral ligament on the joint line on the medial side of the knee and the popliteal groove on the lateral side of the knee (this marker placement is taken from that developed in the CP Cluster Protocol for 3D Marker placement within the CAMARC II EU Programme (1992) with the kinematic modelling methods outlined in Cappozzo 2005 and prior to this by Davis et al 1991). . The long axis of the calf or shank is the line from the knee joint centre to the mid-point of the medial and lateral malleoli. The flexion-extension axis of the ankle is then defined as a line through the lateral malleolus in the plane containing the knee joint centre, the medial and lateral ankle (tips of malleoli) and which is perpendicular to the long axis of the shank (with its medial exit from the ankle typically not through the medial malleolus). This definition is taken from Plug-in-Gait which is a variant of the original model of Davis et al. and has been widely used in clinical gait analysis for many years and continues to be seen as a reference standard. Small surface markers are placed on the patient’s medial and lateral malleoli just prior to digital scanning of the leg. The STL file derived from the scan and used to mill the MDF model contains both the anatomical and technical references required for the alignment in the test rig including the medial end of the plantar/dorsiflexion axis of the ankle.. The long axis of the shank is identified by the midpoint of the ankle reference points and the intersection of 2 lines from medial to lateral and posterior to anterior hemispheres on the mould of the leg just below the knee joint centre (Figure 1A-B) (these points being established by projections from the shank axis of the 3D scan of the leg in the AFO design software).

Davis, Roy B., Õunpuu, Sylvia, Tyburski, Dennis & Gage, James R. A gait analysis data collection and reduction technique. Human Movement Science, 1991,10(5) 575-587

Cappozzo, Aurelio, Della Croce, Ugo ,Leardini, Alberto & Chiari, Lorenzo. Human movement analysis using stereophotogrammetry. Part 1: theoretical background., Gait and Posture 21 (2005) 186–196 Review

C.E.C. Program AIM, Project A-2002: CAMARC-II, 1992.


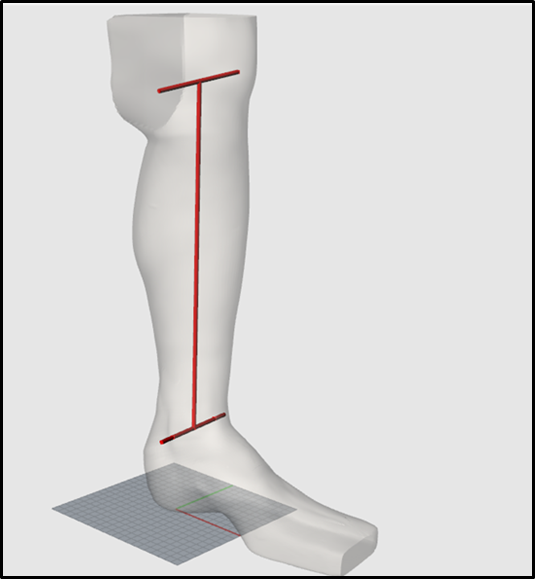

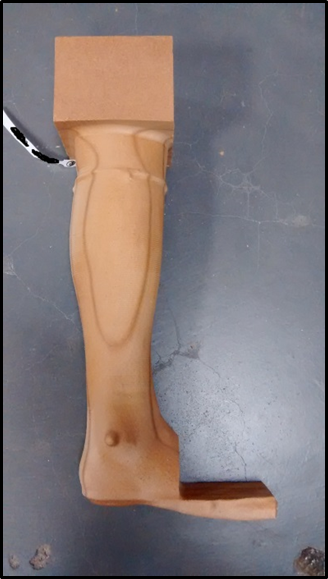


**A**

**B**

**Figure 1A: Drawing of the patient leg model: 1. Ankle axis, 2. Shank axis, 3. Knee joint centre; Figure 1B: Patient leg model milled from MDF: 4. marker on the lateral malleolus for the ankle axis identification; 5. markers on the frontal, lateral, and posterior side for the shank axis identification.**
