# Supplementary material for: A novel experimental setup for evaluating the stiffness of ankle foot orthoses
Source: BMC Res Notes. 2018 Sep 5;11:649. doi: 10.1186/s13104-018-3752-4 (PMC6125880; doi:10.1186/s13104-018-3752-4)
Supplement: Supplementary file 2 — Additional file 2. Detail of the experimental setup. [file 13104_2018_3752_MOESM2_ESM.docx]

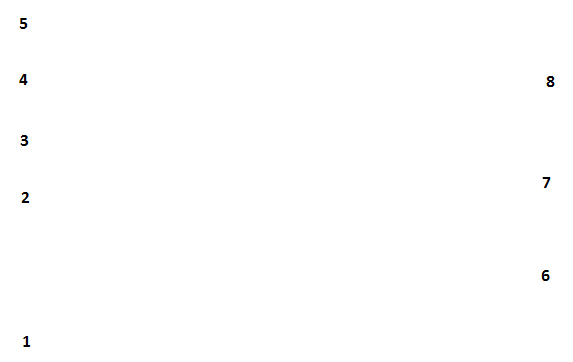

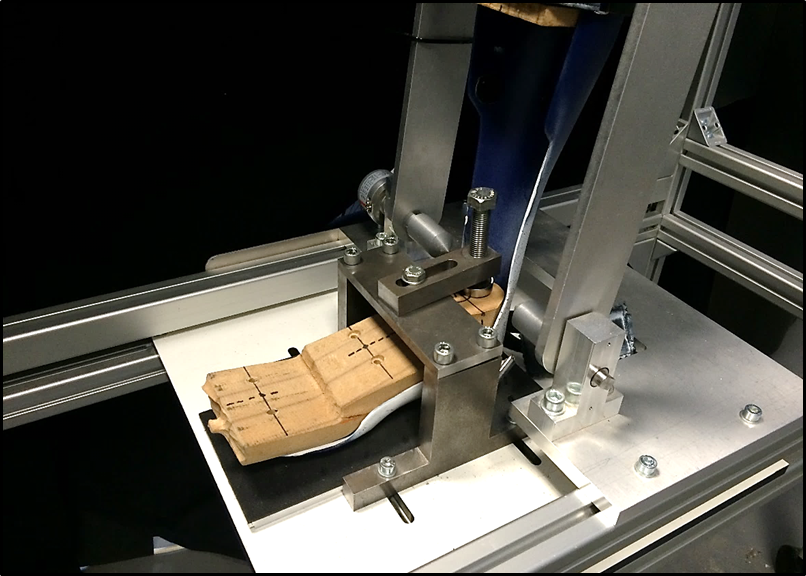


**Figure 2: Detail of the experimental setup: 1. MDF foot block; 2. Clamping system; 3. Pointer for the ankle axis alignment; 4. Incremental optical encoder; 5. Compression screw; 6. Ankle rotation axis ; 7. U-shaped frame; 8. AFO.**
