# Supplementary material for: A novel experimental setup for evaluating the stiffness of ankle foot orthoses
Source: BMC Res Notes. 2018 Sep 5;11:649. doi: 10.1186/s13104-018-3752-4 (PMC6125880; doi:10.1186/s13104-018-3752-4)
Supplement: Supplementary file 3 — Additional file 3. Detail of the load cell–linear motor complex. [file 13104_2018_3752_MOESM3_ESM.docx]

**Details of the experimental set up**


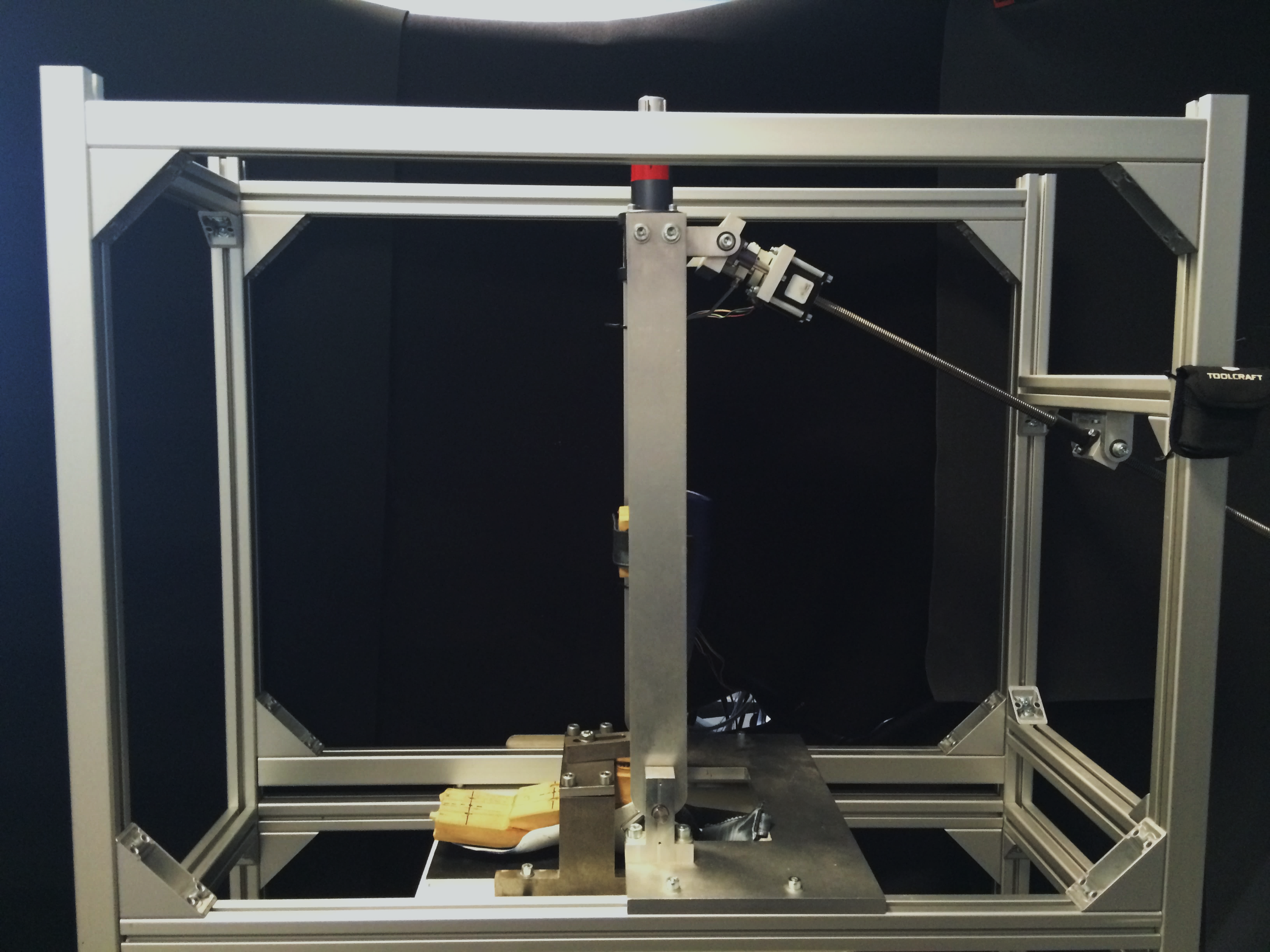


**Figure 3: Detail of the load cell–linear motor complex: 1. Load cell ; 2. Linear motor; 3. Bearing**
