# Supplementary material for: A novel experimental setup for evaluating the stiffness of ankle foot orthoses
Source: BMC Res Notes. 2018 Sep 5;11:649. doi: 10.1186/s13104-018-3752-4 (PMC6125880; doi:10.1186/s13104-018-3752-4)
Supplement: Supplementary file 4 — Additional file 4. Figure of the two experimental settings used to test the ‘CalibrAFO’ device. [file 13104_2018_3752_MOESM4_ESM.docx]

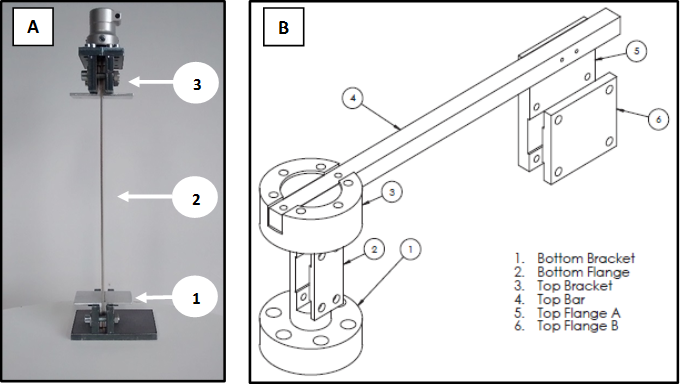


**Figure 4: In 4A the side view of the CalibrAFO:1. bottom clamp; 2. inox steel sheet; 3. top clamp. In 4B the mounting frame for the Instron Electropuls E10000 testing machine.**
