# Supplementary material for: A novel experimental setup for evaluating the stiffness of ankle foot orthoses
Source: BMC Res Notes. 2018 Sep 5;11:649. doi: 10.1186/s13104-018-3752-4 (PMC6125880; doi:10.1186/s13104-018-3752-4)
Supplement: Supplementary file 5 — Additional file 5. Results obtained from the tests on the ‘CalibrAFO’ device. [file 13104_2018_3752_MOESM5_ESM.docx]

**Figure 5: Comparison of the results between the setup described in the article and the Instron Electropuls E10000 testing machine.**
