# Supplementary material for: A novel experimental setup for evaluating the stiffness of ankle foot orthoses
Source: BMC Res Notes. 2018 Sep 5;11:649. doi: 10.1186/s13104-018-3752-4 (PMC6125880; doi:10.1186/s13104-018-3752-4)
Supplement: Supplementary file 6 — Additional file 6. Results in terms of the AFO rig test–retest repeatability. [file 13104_2018_3752_MOESM6_ESM.docx]

**Table 1: AFO rig test-retest repeatability: measures expressed as absolute values and percentage difference from the mean.**

|  |  |  |  |  | **AFO A** |  |  |  |  |  |  |
| --- | --- | --- | --- | --- | --- | --- | --- | --- | --- | --- | --- |
| **Test** | **PL [Nm/°]** | **PL [%]** |  | **PU [Nm/°]** | **PU [%]** |  | **DL [Nm/°]** | **DL [%]** |  | **DU [Nm/°]** | **DU[%]** |
| 1st | 2.96 | 0.56 |  | 3.17 | 0.01 |  | 2.66 | 0.78 |  | 2.63 | 0.68 |
| 2nd | 2.97 | 0.06 |  | 3.16 | 0.21 |  | 2.64 | 0.01 |  | 2.60 | 0.59 |
| 3rd | 2.99 | 0.62 |  | 3.18 | 0.20 |  | 2.62 | 0.77 |  | 2.61 | 0.09 |
| Mean | 2.97 |  |  | 3.17 |  |  | 2.64 |  |  | 2.62 |  |
| SD | 0.02 |  |  | 0.01 |  |  | 0.02 |  |  | 0.02 |  |
|  |  |  |  |  | **AFO B** |  |  |  |  |  |  |
| **Test** | **PL [Nm/°]** | **PL [%]** |  | **PU [Nm/°]** | **PU [%]** |  | **DL [Nm/°]** | **DL [%]** |  | **DU [Nm/°]** | **DU[%]** |
| 1st | 3.41 | 0.27 |  | 3.08 | 0.25 |  | 2.87 | 0.16 |  | 2.74 | 0.40 |
| 2nd | 3.43 | 0.16 |  | 3.07 | 0.03 |  | 2.84 | 0.74 |  | 2.74 | 0.71 |
| 3rd | 3.43 | 0.11 |  | 3.06 | 0.29 |  | 2.88 | 0.58 |  | 2.69 | 1.11 |
| Mean | 3.42 |  |  | 3.07 |  |  | 2.86 |  |  | 2.72 |  |
| SD | 0.01 |  |  | 0.01 |  |  | 0.02 |  |  | 0.03 |  |
|  |  |  |  |  | **AFO C** |  |  |  |  |  |  |
| **Test** | **PL [Nm/°]** | **PL [%]** |  | **PU [Nm/°]** | **PU [%]** |  | **DL [Nm/°]** | **DL [%]** |  | **DU [Nm/°]** | **DU[%]** |
| 1st | 3.57 | 0.96 |  | 3.50 | 1.26 |  | 2.93 | 1.33 |  | 2.82 | 1.58 |
| 2nd | 3.54 | 0.26 |  | 3.48 | 0.74 |  | 2.88 | 0.21 |  | 2.77 | 0.17 |
| 3rd | 3.49 | 1.22 |  | 3.38 | 2 |  | 2.86 | 1.12 |  | 2.73 | 1.41 |
| Mean | 3.53 |  |  | 3.45 |  |  | 2.89 |  |  | 2.77 |  |
| SD | 0.04 |  |  | 0.06 |  |  | 0.04 |  |  | 0.04 |  |
|  |  |  |  |  | **AFO D** |  |  |  |  |  |  |
| **Test** | **PL [Nm/°]** | **PL [%]** |  | **PU [Nm/°]** | **PU [%]** |  | **DL [Nm/°]** | **DL [%]** |  | **DU [Nm/°]** | **DU[%]** |
| 1st | 6.16 | 0.13 |  | 5.58 | 0.60 |  | 3.97 | 0.02 |  | 3.56 | 0.87 |
| 2nd | 6.17 | 0.02 |  | 5.53 | 0.25 |  | 3.97 | 0.05 |  | 3.52 | 0.30 |
| 3rd | 6.17 | 0.10 |  | 5.52 | 0.35 |  | 3.97 | 0.07 |  | 3.51 | 0.57 |
| Mean | 6.17 |  |  | 5.54 |  |  | 3.97 |  |  | 3.53 |  |
| SD | 0.01 |  |  | 0.03 |  |  | 0.01 |  |  | 0.03 |  |
