# Supplementary material for: A novel experimental setup for evaluating the stiffness of ankle foot orthoses
Source: BMC Res Notes. 2018 Sep 5;11:649. doi: 10.1186/s13104-018-3752-4 (PMC6125880; doi:10.1186/s13104-018-3752-4)
Supplement: Supplementary file 7 — Additional file 7. Results in terms of the AFO rig intra-tester variability. [file 13104_2018_3752_MOESM7_ESM.docx]

**Table 2: Intra-tester variability: measures expressed as absolute values and percentage difference from the mean.**

|  |  |  |  |  | **AFO A** |  |  |  |  |  |  |
| --- | --- | --- | --- | --- | --- | --- | --- | --- | --- | --- | --- |
| **Test** | **PL [Nm/°]** | **PL [%]** |  | **PU [Nm/°]** | **PU [%]** |  | **DL [Nm/°]** | **DL [%]** |  | **DU [Nm/°]** | **DU[%]** |
| 1st | 2.96 | 0.61 |  | 3.17 | 4.20 |  | 2.66 | 1.81 |  | 2.63 | 3.96 |
| 2nd | 2.92 | 0.61 |  | 3.45 | 4.20 |  | 2.56 | 1.81 |  | 2.43 | 3.96 |
| Mean | 2.94 |  |  | 3.31 |  |  | 2.61 |  |  | 2.53 |  |
|  | 0.03 |  |  | 0.20 |  |  | 0.07 |  |  | 0.14 |  |
|  |  |  |  |  | **AFO B** |  |  |  |  |  |  |
| **Test** | **PL [Nm/°]** | **PL [%]** |  | **PU [Nm/°]** | **PU [%]** |  | **DL [Nm/°]** | **DL [%]** |  | **DU [Nm/°]** | **DU[%]** |
| 1st | 3.41 | 1.30 |  | 3.08 | 2.83 |  | 2.87 | 1.16 |  | 2.74 | 0.51 |
| 2nd | 3.33 | 1.30 |  | 2.91 | 2.83 |  | 2.93 | 1.16 |  | 2.76 | 0.51 |
| Mean | 3.37 |  |  | 2.99 |  |  | 2.90 |  |  | 2.75 |  |
| SD | 0.06 |  |  | 0.12 |  |  | 0.05 |  |  | 0.02 |  |
|  |  |  |  |  | **AFO C** |  |  |  |  |  |  |
| **Test** | **PL [Nm/°]** | **PL [%]** |  | **PU [Nm/°]** | **PU [%]** |  | **DL [Nm/°]** | **DL [%]** |  | **DU [Nm/°]** | **DU[%]** |
| 1st | 3.57 | 5.26 |  | 3.50 | 3.42 |  | 2.93 | 3.87 |  | 2.82 | 0.21 |
| 2nd | 3.96 | 5.26 |  | 3.74 | 3.42 |  | 2.71 | 3.87 |  | 2.80 | 0.21 |
| Mean | 3.76 |  |  | 3.62 |  |  | 2.82 |  |  | 2.81 |  |
| SD | 0.28 |  |  | 0.18 |  |  | 0.15 |  |  | 0.01 |  |
|  |  |  |  |  | **AFO D** |  |  |  |  |  |  |
| **Test** | **PL [Nm/°]** | **PL [%]** |  | **PU [Nm/°]** | **PU [%]** |  | **DL [Nm/°]** | **DL [%]** |  | **DU [Nm/°]** | **DU[%]** |
| 1st | 6.16 | 0.43 |  | 5.58 | 3.33 |  | 3.97 | 1.59 |  | 3.56 | 3.98 |
| 2nd | 6.21 | 0.43 |  | 5.96 | 3.33 |  | 3.84 | 1.59 |  | 3.85 | 3.98 |
| Mean | 6.19 |  |  | 5.77 |  |  | 3.91 |  |  | 3.71 |  |
| SD | 0.04 |  |  | 0.27 |  |  | 0.09 |  |  | 0.21 |  |
