# Supplementary material for: A novel experimental setup for evaluating the stiffness of ankle foot orthoses
Source: BMC Res Notes. 2018 Sep 5;11:649. doi: 10.1186/s13104-018-3752-4 (PMC6125880; doi:10.1186/s13104-018-3752-4)
Supplement: Supplementary file 8 — Additional file 8. Results in terms of the AFO rig inter-tester variability. [file 13104_2018_3752_MOESM8_ESM.docx]

**Table 3: Inter-tester variability: measures expressed as absolute values and percentage difference from the mean.**

|  |  |  |  |  | **AFO A** |  |  |  |  |  |  |
| --- | --- | --- | --- | --- | --- | --- | --- | --- | --- | --- | --- |
| **Test** | **PL [Nm/°]** | **PL [%]** |  | **PU [Nm/°]** | **PU [%]** |  | **DL [Nm/°]** | **DL [%]** |  | **DU [Nm/°]** | **DU[%]** |
| 1st | 2.96 | 1.07 |  | 3.17 | 2.74 |  | 2.66 | 5.65 |  | 2.63 | 0.97 |
| 2nd | 3.02 | 1.07 |  | 3.35 | 2.74 |  | 2.98 | 5.65 |  | 2.58 | 0.97 |
| Mean | 2.99 |  |  | 3.26 |  |  | 2.82 |  |  | 2.61 |  |
| SD | 0.05 |  |  | 0.13 |  |  | 0.23 |  |  | 0.04 |  |
|  |  |  |  |  | **AFO B** |  |  |  |  |  |  |
| **Test** | **PL [Nm/°]** | **PL [%]** |  | **PU [Nm/°]** | **PU [%]** |  | **DL [Nm/°]** | **DL [%]** |  | **DU [Nm/°]** | **DU[%]** |
| 1st | 3.41 | 2.28 |  | 3.08 | 1.92 |  | 2.87 | 1.97 |  | 2.74 | 1.91 |
| 2nd | 3.57 | 2.28 |  | 3.20 | 1.92 |  | 2.98 | 1.97 |  | 2.84 | 1.91 |
| Mean | 3.49 |  |  | 3.14 |  |  | 2.92 |  |  | 2.79 |  |
| SD | 0.11 |  |  | 0.09 |  |  | 0.08 |  |  | 0.08 |  |
|  |  |  |  |  | **AFO C** |  |  |  |  |  |  |
| **Test** | **PL [Nm/°]** | **PL [%]** |  | **PU [Nm/°]** | **PU [%]** |  | **DL [Nm/°]** | **DL [%]** |  | **DU [Nm/°]** | **DU[%]** |
| 1st | 3.57 | 1.04 |  | 3.50 | 3.70 |  | 2.93 | 1.50 |  | 2.82 | 0.97 |
| 2nd | 3.64 | 1.04 |  | 3.25 | 3.70 |  | 2.84 | 1.50 |  | 2.76 | 0.97 |
| Mean | 3.60 |  |  | 3.37 |  |  | 2.88 |  |  | 2.79 |  |
| SD | 0.05 |  |  | 0.18 |  |  | 0.06 |  |  | 0.04 |  |
|  |  |  |  |  | **AFO D** |  |  |  |  |  |  |
| **Test** | **PL [Nm/°]** | **PL [%]** |  | **PU [Nm/°]** | **PU [%]** |  | **DL [Nm/°]** | **DL [%]** |  | **DU [Nm/°]** | **DU[%]** |
| 1st | 6.16 | 1.63 |  | 5.58 | 2.14 |  | 3.97 | 0.91 |  | 3.56 | 1.37 |
| 2nd | 5.96 | 1.63 |  | 5.34 | 2.14 |  | 3.90 | 0.91 |  | 3.66 | 1.37 |
| Mean | 6.06 |  |  | 5.46 |  |  | 3.93 |  |  | 3.61 |  |
| SD | 0.14 |  |  | 0.16 |  |  | 0.05 |  |  | 0.07 |  |
